# Nanoscale spheroidized cementite induced ultrahigh strength-ductility combination in innovatively processed ultrafine-grained low alloy medium-carbon steel

**DOI:** 10.1038/s41598-017-02920-9

**Published:** 2017-06-02

**Authors:** N. Jia, Y. F. Shen, J. W. Liang, X. W. Feng, H. B. Wang, R. D. K. Misra

**Affiliations:** 1 Key Laboratory for Anisotropy and Texture of Materials (Ministry of Education), School of Material Science and Engineering, Northeastern University, Shenyang, 110819 China; 2 General Engineering Research Institute of Chinese Academy of Engineering Physics, Mianyang, 621999 China; 3 Department of Metallurgical, Materials and Biomedical Engineering, University of Texas at El Paso, 500 W University Avenue, El Paso, 79968 USA

## Abstract

We describe here innovative processing of low alloy medium-carbon steel with a duplex microstructure composed of nanoscale spheroidized cementite (Fe_3_C) in an ultrafine-grained (UFG) ferritic steel. After multi-pass rolling and intermittent annealing at 550 °C for 300 s, the obtained UFG-1 steel showed an average ferrite grain size of ~430 nm, containing nanoscale spheroidized cementite (Fe_3_C) particles with an average size of ~70 nm. On annealing at 600 °C for 300 s, the average size of ferritic grains was increased to ~680 nm and the average size of spheroidized Fe_3_C particles increased to ~90 nm, referred as UFG-2 steel. Tensile tests indicated that UFG-1 steel had high yield strength (*σ*_y_) of 1260 MPa, and ultimate tensile strength (*σ*_UTS_) of 1400 MPa. These values are higher than that of UFG-2 steel (*σ*_y_ = 1080 MPa and *σ*_UTS_ = 1200 MPa), suggesting that the strengthening contribution is a cumulative effect of decrease in ferrite grain size and nanoscale cementite. The incoherent interfaces between nanosized particles and the matrix acted as a strong barrier to dislocation motion. The study underscores that nanosized precipitates not only provide strength but also contribute to ductility, which is very encouraging for improving the ductility of medium-carbon steels.

## Introduction

Metals and alloys produced by severe plastic deformation (SPD) techniques such as equal channel angular pressing (ECAP)^1^, severe-plastic torsion straining (SPTS)^2^, and heavy deformation by rolling, drawing, and ball-milling^3–5^ are attractive for obtaining superior room temperature strength^1–7^. Materials processed by these methods are characterized by a microstructure mainly consisting of ultrafine grains (UFG) that increases hardness and strength. Grain refinement of steels results in a decrease of ductile-to-brittle transition temperature^8, 9^.

Grain refinement may not improve the ductility of UFG materials, but inherently poor ductility can be improved by second phase particles, through accumulation of geometrically necessary dislocations around the particles^10^. The spheroidized microstructure consisting of plate-type lamellar cementite and ferrite matrix provides lower hardness but higher ductility than the pearlite microstructure^11^. On the other hand, spheroidized carbide particles embedded in ferrite matrix contribute to high ductility^12, 13^. The superior ductility and low hardness of the spheroidized microstructure play an important role in the subsequent cold forming of low-carbon steels.

The D6AC steel belongs to medium-carbon steels with low alloying content. The carbon content is in the range of 0.42–0.48 wt.% together with other alloying elements in accordance to ASTM standard 6431 (i.e., 0.9–1.2 Cr, 0.9–1.1 Mo, 0.4–0.7 Ni, 0.08–0.15 V, 0.6–0.9 Mn, and 0.15–0.3 Si, in wt.%). This group of steels has high strength and large yield-to-tensile strength ratio, and consequently is widely used as pressure vessels, aerospace and defense components^14–16^. However, the application of these steels is limited by their poor ductility at high strength level^17, 18^. Several studies on the fracture of D6AC steel have been conducted^19, 20^. Mechanical properties of D6AC may change considerably when the impact of the subsequent annealing treatment is reduced^21–23^. To improve the ductility and formability of steels, a typical heat-treatment process for D6AC steel that has been suggested is quenching from a high austenitic temperature to room temperature, followed by conventional tempering^11^. This process can be referred as spheroidization treatment, because spheroidized carbide particles (i.e., cementite or Fe_3_C) are expected to be uniformly distributed in the ferrite matrix. These spheroidized Fe_3_C particles have a potential to improve the ductility of this class of steels. The spheroidized microstructure is formed at an appropriate temperature when the time for carbon diffusion reaction is adequate. However, the spheroidization of cementite can influence the mechanical properties of steels, such as leading to a reduction in hardness with slightly improved ductility. This is because the spherical cementite is softer than the lamellar counterpart^7, 24, 25^. The microstructure consisting of spheroidized cementite particles in an ultrafine ferrite matrix can be obtained through conventional cold or warm rolling plus subsequent annealing in low-carbon steels^6, 7, 26^. While the processing route and mechanical properties of ultrafine-grained steels have been previously reported, corresponding studies on medium-carbon steels have rarely been conducted because of difficulties experienced in controlling the microstructure during spheroidization annealing. First, the rate of spheroidization is influenced by carbon diffusion in ferrite. Second, the addition of a strong carbide forming element such as Ti, V, and Nb decreases the diffusivity of carbon in ferrite, such that the spheroidization reaction becomes slow. Third, the small cementite particles grow or coalesce to minimize the total interfacial surface area at a given temperature, resulting in coarse particles^11^. Lastly, the spheroidization process is related to the matrix and phase composition that are determined by hot rolling parameters including rolling temperature, number of passes, strain rate, etc.

The objective of the study described here is threefold: (i) To spheroidize cementite to nanoscale in a high strength low-alloy (HSLA) steel of chemical composition, Fe-1.05Cr-1.01Mo-0.73Mn-0.61Ni-0.17Si-0.09V-0.43C (wt.%) through an innovative process. (ii) To refine the ferrite matrix to ultrafine-grained regime. (iii) To quantify the effect of nanoscale Fe_3_C particles on the mechanical properties of low-alloyed steel. To accomplish the objectives and to elucidate the relationship between structure and mechanical properties, uniaxial tensile tests together with post-mortem transmission electron microscopy (TEM) was carried out. The study provides new insights into the micromechanisms contributing to work hardening of HSLA steels. It is expected that the present study is beneficial in the design of medium- and high-carbon steels with nanosized composite structures.

## Experimental procedure

### Materials

The alloy was melted in a vacuum induction furnace, and an ingot of dimension 300 × 200 × 20 mm^3^ was cast. The chemical composition of the alloy, measured by inductively coupled plasma mass spectroscopy was as follows: 1.05Cr, 1.01Mo, 0.73Mn, 0.61Ni, 0.17Si, 0.09 V, 0.43 C (in wt.%), and balance Fe. A plate was cut from the ingot, homogenized in an air furnace at 1200 °C for 2 hours to remove the inhomogeneous microstructure evolved during solidification, hot rolled at 1000 °C (ensure complete dissolution of carbide particles present in the initial condition) via 9 passes to thickness of 6.5 mm (ε = 85%), and then quenched to room temperature in water (at a cooling rate of ~50 °C/s) to obtain a fully martensitic microstructure. Subsequently, the plate was divided into two parts and annealed at 750 °C for 300 s (martensite transformed to ferrite). Following warm rolling at 750 °C for 6 passes with ~12% reduction per pass, a final thickness of 1.5 mm was obtained. Two intermittent annealing treatments, i.e., annealing at 550 °C and 600 °C for 300 s after each pass, were performed for the two parts, respectively. Our aim was to obtain ultrafine-grained (UFG) microstructure. Based on the applied annealing temperature, the steel annealed at 550 °C and 600 °C are referred as UFG-1 and UFG-2, respectively.

## Microstructural characterization

### X-ray diffraction

To determine phases, X-ray diffraction measurements were performed on the surface of the rolled plates, i.e., the RD-TD planes (RD: rolling direction, TD: transverse direction). The Rigaku Smart-Lab diffractometer equipped with a sample stage in which a Co anode (wavelength *k* = 0.1789 nm) and an X-Celebrator detector was used. The penetration depth of X-rays (Co radiation) into steels was a few micrometers.

### Fine-scale characterization

Using scanning electron microscopy (SEM) in conjunction with electron backscatter diffraction (EBSD), the grain morphology of the as-processed steels was characterized. The RD-TD planes of the specimens were mechanically polished and finally electropolished to remove the surface damage induced by grinding and mechanical polishing. The electrolyte consisted of 10 vol. % perchloric acid and 90 vol. % ethanol. The EBSD data were collected using a SU-70 Hitachi field-emission SEM at an accelerated voltage of 20 kV and a sample tilt angle of 70°. The selected scanning step was 0.1 µm, and the analyzed area was 1.5 × 1.0 mm^2^ for each specimen. Three specimens in each condition were analyzed and the acquired data were evaluated using the TSL software.

Specimens for TEM studies were cut from the homogeneously deformed regions (uniform elongated zone) of the tensile specimens. The specimens were mechanically polished from both sides to a thickness of ~60 µm. Then, the foils were thinned using a double-jet electrolytic polisher at a voltage of 32 V and temperature in the range of −10 and −5 °C. TEM studies on the strained samples were performed in a Tecnai G2 20 microscope, operated at an accelerated voltage of 200 kV.

### Tensile tests

The dog-bone shaped specimens were cut from the middle of the as-annealed steel along RD using electron discharge machining, and mechanically polished using silica paper to dimensions of 15 (gauge length) × 3 (gauge width) × 1.5 (gauge thickness) mm^3^. The uniaxial tensile tests were conducted using a CMT 5105 pc-controlled mechanical testing system (MTS Co. Ltd, USA) at a constant strain rate of 5 × 10^−3^ s^−1^ at room temperature. During loading, an extensometer was used to calibrate and measure the strain.

## Results and Discussion

**Figure 1:**
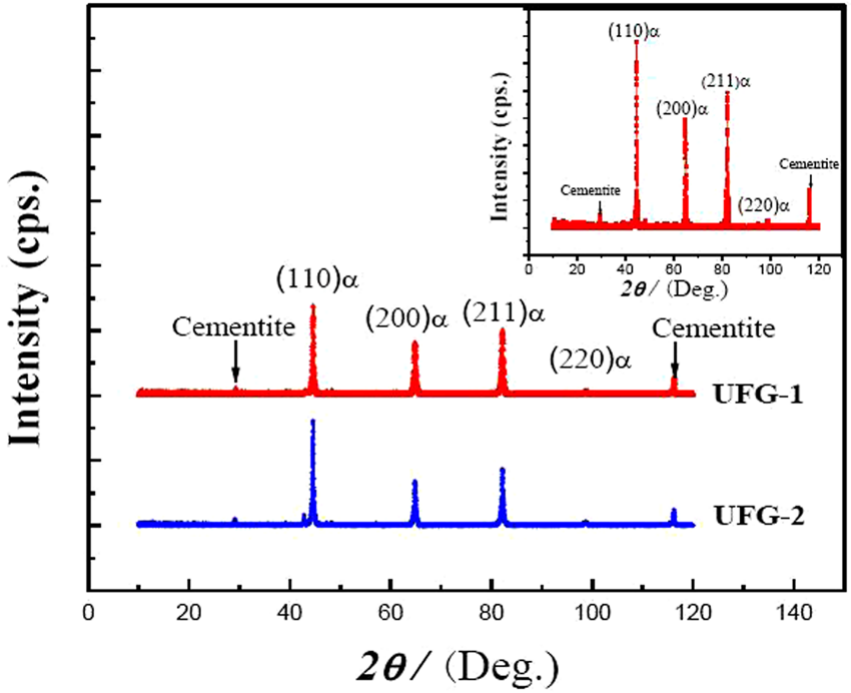
XRD patterns of experimental steels with chemical composition of 1.05Cr-1.01Mo-0.73Mn-0.61Ni-0.17Si-0.09V-0.43 C, in wt.%. The upper right inset shows the magnified pattern of UFG-2 steel.

Figure 1 shows X-ray diffraction patterns of low alloy medium-carbon steels with two different grain sizes (UFG-1 and UFG-2). It is evident that the both steels consist of bcc phase and carbide (cementite). The upper right inset shows the magnified pattern for UFG-2 steel, in which the arrows indicate peaks corresponding to the occurrence of cementite. By comparing the measurements with the standard powder diffraction patterns, the existence of cementite is verified by the weak peaks at 29.2° and 116.5° for the both steels, whilst the peaks at 44.7°, 64.9°, 82.2°, and 98.7° correspond to the (110), (200), (211) and (220) diffraction planes of the ferrite matrix, respectively. Different peak intensities indicate the difference not only in grain size but also orientation distribution between the two specimens. For example, the intensities of (200) and (211) peaks in the UFG-1 steel are stronger than that in the UFG-2 steel. However, the (110) peak is stronger in the UFG-2 compared with the UFG-1 steel.

**Figure 2:**
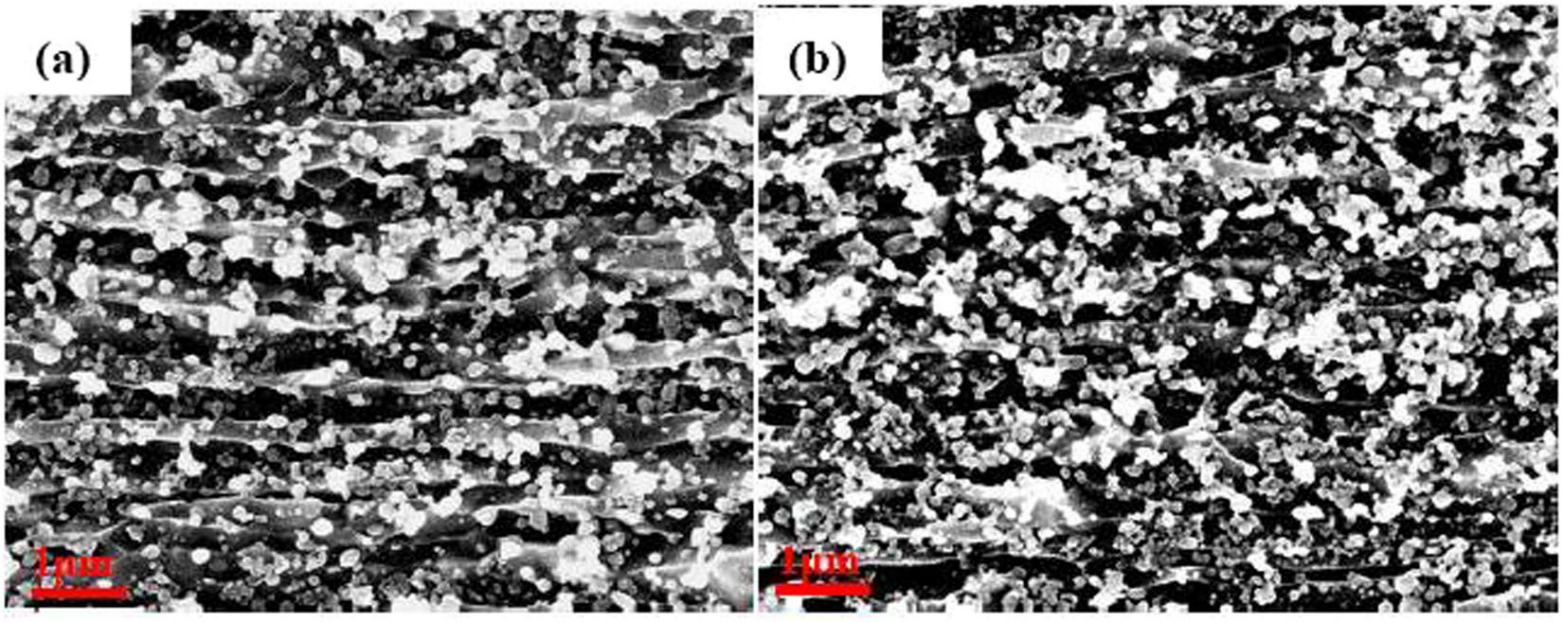
Scanning electron micrographs of (**a**) UFG-1 and (**b**) UFG-2 steels.

**Figure 3:**
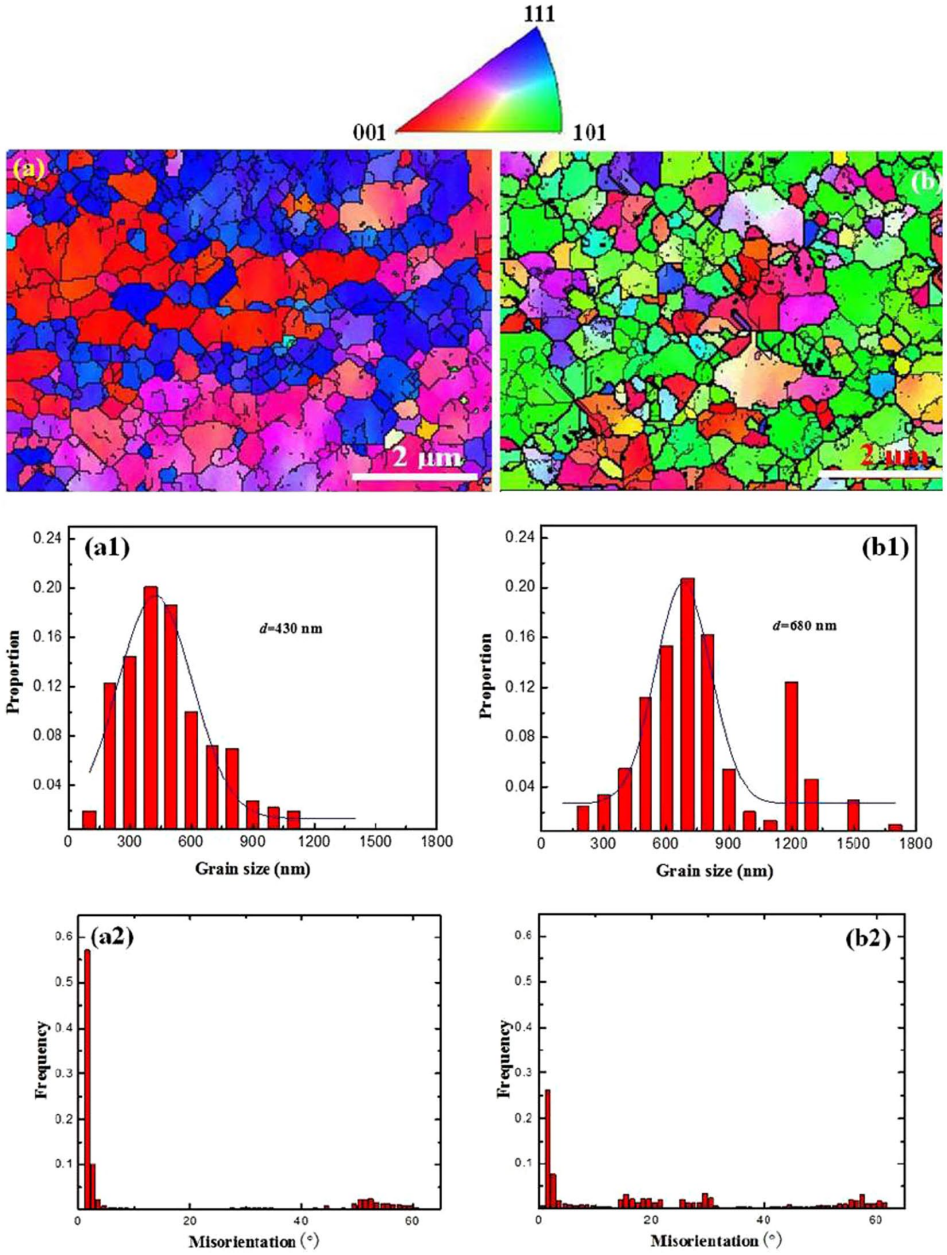
EBSD orientation maps for (**a**) UFG-1and (**b**) UFG-2 steels. Statistical grain size for (a1) UFG-1and (b1) UFG-2 steels. The average grain size is ~430 nm and ~680 nm for UFG-1 and UFG-2 steels, respectively. Histogram of misorientation for (a2) UFG-1 and (b2) UFG-2 steels.

To explore microstructural evolution in UFG steels obtained from different heat-treatment processes, observations were made by SEM. Figure 2 shows that in both UFG-1 and UFG-2 steels, numerous white particles coexist within the gray ferrite matrix. This indicates that the combination of warm rolling and intermittent annealing process promotes not only the precipitation of carbides but also refinement of ferrite. With increased annealing temperature to 600 °C, the ferrite is transformed from strip-like (resulting from incomplete recrystallization and recovery) (Fig. 2a) to equiaxed morphology resulting from recrystallization (Fig. 2b), accompanied by an increase in the carbide particle size. The EBSD characterization of both the steels clearly reveals a recrystallized microstructure, which is evident by the presence of equiaxed grains (Fig. 3a,b). As shown in the typical orientation maps from EBSD, the as-annealed UFG-1 steel consists of grains with significant <111>//ND and <001>//ND orientations (Fig. 3a). This result is similar to that reported for annealed and rolled ferritic steels^27^. For as-annealed UFG-2 steel, the dominant orientation is <101>//ND (Fig. 3b), which is an indication that the steel is mainly composed of recrystallized grains. To achieve good ductility, {111} <uvw> texture is more beneficial than {110} <uvw> for bcc steels^28^. Obviously, here a lower annealing temperature of 550 °C is useful to obtain {111} <uvw> texture. We can see that the orientation distribution obtained from EBSD analysis is consistent with the XRD results. In Fig. 1, the (110) peak is higher whilst the (100) peak is slightly lower in the UFG-2 steel in comparison with the corresponding peaks in the UFG-1 steel.

On the other hand, the EBSD analysis showed that the average grain size is ~430 nm and ~680 nm for UFG-1 (Fig. 3a1) and UFG-2 (Fig. 3b1) steels, respectively. For UFG-1 steel, the fraction of low angle grain boundaries (with grain boundary misorientations <15°) is as high as 75% (Fig. 3a2). A large fraction of low angle grain boundaries indicates that grains are not real grains but many subgrains are present in this steel. However, in UFG-2 steel, the fraction of high-angle grain boundaries (HAGBs) (with grain boundary misorientations ≥15°) is as high as ~60%, indicating that a higher annealing temperature of 600 °C is beneficial for recrystallization of ferritic grains in this steel (Fig. 3b2). Additionally, one could see a few large grains (in the right lower corner, grain diameter larger than 2 μm) in two steels, which resulted from abnormal growth of grains. EBSD grain size obtained from ~150 grains for each steels are appropriate. The indexing ratio for the images is 89%, hence artifacts present inside some grains (incomplete pieces of “grain boundaries”, Fig. 3a and b). According to the EBSD results, it is reasonable to deduce that the UFG-1 steel should exhibit a higher strength than the UFG-2 steel. The strengthening effect related to the grain refinement can be represented by the classical Hall-Petch relationship^29^, i.e., *σ*_*y*_ = *σ*_*0*_ + *K*·*d* ^−*0.5*^, where *σ*_*0*_ is friction stress, *K* is a constant and *d* is the mean grain size.

**Figure 4:**
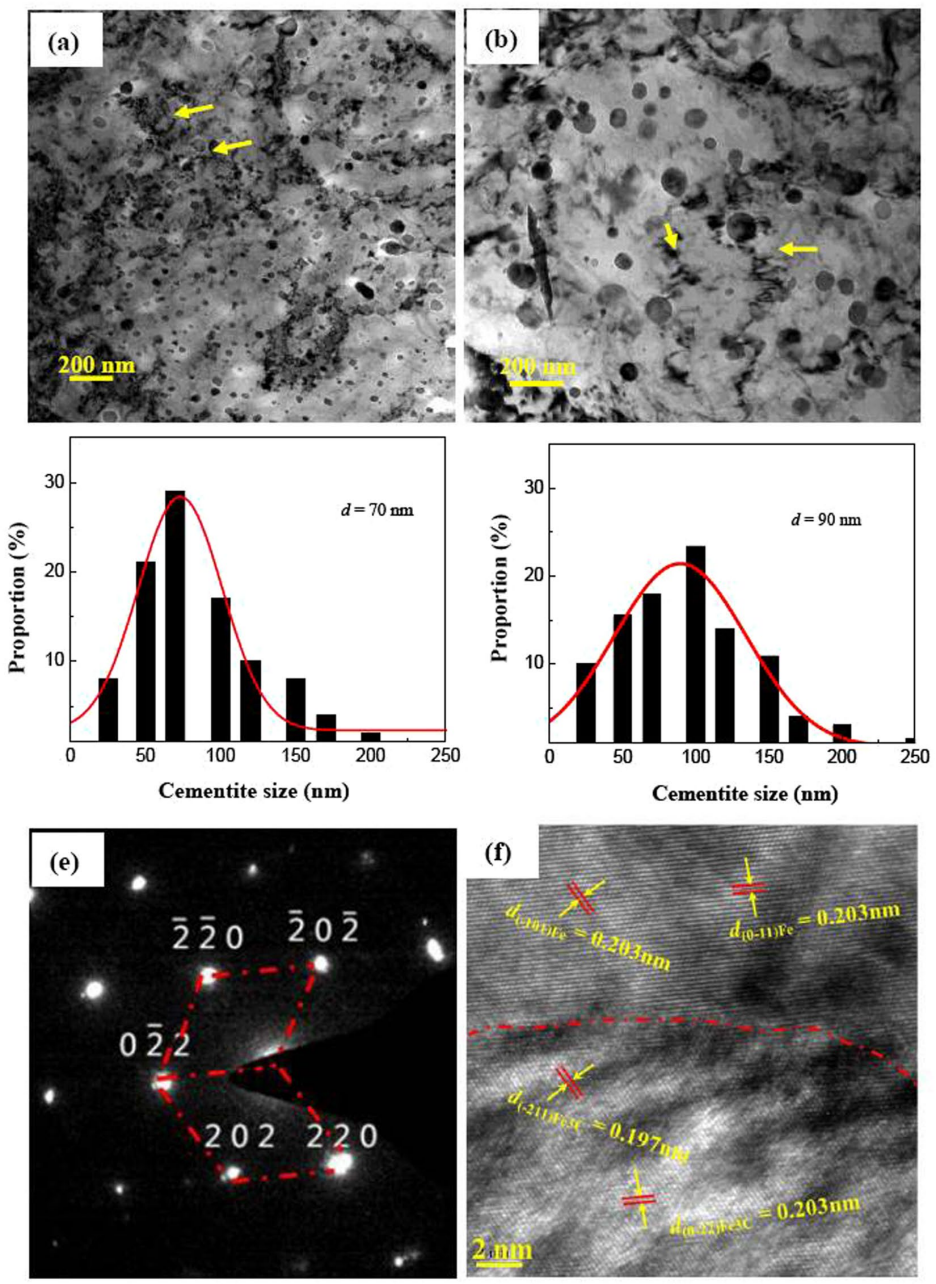
TEM micrographs of (**a**) UFG-1 and (**b**) UFG-2 steels observed along the ${[010]}_{{\rm{\alpha }}}$ zonal axis. (**e**) Shows selected-area-electron-diffraction (SAED) patterns of Fe_3_C particles. (**f**) Is the high-resolution TEM (HRTEM) image of cementite and ferrite. UFG-1 steel: ferrite (*D* = 430 nm) + spheroidized cementite (*d* = 70 nm); UFG-2 steel: ferrite (*D* = 680 nm) + spheroidized cementite (*d* = 90 nm).

TEM studies were carried out to explore microstructural features of UFG steels. The underlying reason is that SEM/EBSD technique cannot distinguish the size of nanoscale particles. Figure 4a and b show representative micrographs illustrating typical microstructural characteristics of UFG-1 and UFG-2 steels. The size of nanoscale particles in the steels increased with increased annealing temperature, which is consistent with SEM observations. In both steels, most of the nanoparticles are spherical and a few particles (as indicated by arrows) are located at the grain boundaries (as indicated by broken lines). The formation of spherical particles is envisaged to be associated with the initial shape of the particles in tempered martensite. This spheroidized microstructure should be stable because of stress relief in the ferritic matrix and minimized interfacial surface area per unit volume between the cementite particle and the ferrite^11^.

Based on TEM observations, the size of Fe_3_C particles measured and the statistical data is summarized in Fig. 4c and d for UFG-1 and UFG-2 steels, respectively. Average size of the particles increases from 70 nm in UFG-1 steel to 90 nm in UFG-2 steel, corresponding to a volume fraction (*f*_v_) of 10.5% and 7.9% (areas measured from Fig. 4a and b), respectively. Therefore, the ratio of volume fraction (*F*_*v*_) to diameter (*d)* of the precipitates, i.e., *F*_*v*_/*d*, is 1.5 × 10^−3^ nm^−1^ and 8.8 × 10^−4 ^nm^−1^ for the UFG-1 and UFG-2 steels. Detailed TEM characterization for UFG-2 specimens reveals that the diffraction pattern of Fe_3_C particles overlaps with the surrounding ferrite matrix (the Pitsch-Petch orientation relationship^30^ is fulfilled between the cementite and the ferrite). Clearly, cementite is incoherent with the ferrite matrix because the spacing of the neighboring $(\bar{2}11)$ planes in the cementite is 0.197 nm, while that of neighboring $(\bar{1}01)$ planes in the ferrite is 0.203 nm.

**Figure 5:**
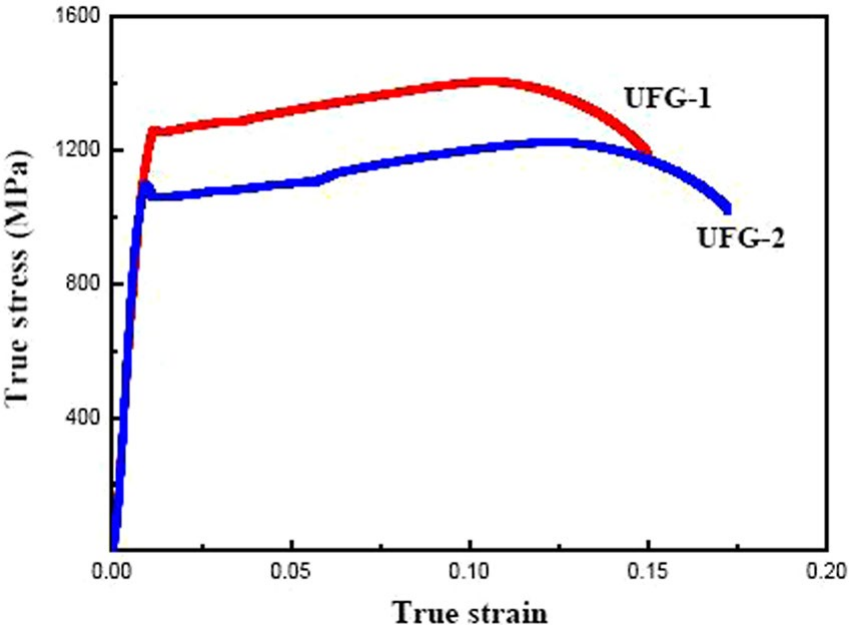
True stress-strain curves for UFG-1 and UFG-2 steels tensile tested at room-temperature.

Figure 5 shows true stress-strain curves for the studied steels at a strain rate of 0.005 s^−1^. Mechanical properties vary with grain size and phase composition. The ultrafine-grained samples exhibit high 0.2% off-set yield stress (*σ*_y_), ultimate-tensile stress (*σ*_UTS_) and elongation-to-failure (ɛ_*f*_). For the UFG-1 steel, *σ*_y_ is 1260 MPa, and *σ*_UTS_ is 1400 MPa. These values are significantly higher than UFG-2 steel (*σ*_y_ = 1080 MPa and *σ*_UTS_ = 1200 MPa). The UFG-1 steel shows an elongation of 15%. This gives the product of strength and elongation value of 21000 MPa%. The product of strength and elongation is an important indicator to assert if a metallic material has good formability. Furthermore, ɛ_*f*_ of UFG-2 steel is 17%, resulting in the product of strength and elongation of 20400 MPa%. In a medium-carbon steel composed of cementite (*d* ~ 610 nm) in fine-grained ferrite (*d* ~ 3.3 μm), *σ*_y_ and *σ*_UTS_ are only 425 MPa and 645 MPa, respectively^31^, which are significantly lower than our UFG steels. In the reported spheroidized D6AC steel of similar chemical composition, even though the ductility is marginally greater at 21%, the *σ*_y_ and *σ*_UTS_ values are only 870 MPa and 930 MPa, respectively^11^. To clearly see work hardening for both UFG steels over a wide strain range, especially at large strains and high stresses. The high strain hardening capacity of the UFG-1 and UFG-2 steels is essential for achieving large uniform elongation without pronounced strain localization such as necking^32^. The remarkable difference in mechanical properties between the currently studied steels and those in literatures should be associated with the significantly different microstructures of the steels. As far as the medium-carbon steel^32^ is concerned, the sizes of both spherical Fe_3_C and ferritic grains are about one order of magnitude larger than that in the UFG-1 steel, i.e., 610 nm vs. 70 nm for cementite and 3300 nm vs. 430 nm for ferrite, respectively. On the other hand, the large elongation and low strength in the spheroidized D6AC steel^11^ must be resulted from the coarse ferritic grains or bainitic laths (several microns), despite of the existence of nanosized cementite particles.

The results can be explained by the different strain hardening behavior of the constituent phases as well as the differences in grain size in the two studied steels. The good combination of strength and ductility in the both UFG steels is associated with different contribution from the duplex microstructure consisting of soft ferrite matrix and hard Fe_3_C particles. The decrease in ferrite grain size and nanoscale particles are responsible for high *σ*_y_ and *σ*_UTS_ of UFG-1 steel. The coarsening of spheroidized carbide particles also leads to reduction of *σ*_y_ and *σ*_UTS._ On the other hand, the elongation can be compensated by increasing ferrite grain size and coarsening of Fe_3_C particles.

Several studies suggested that nanosized precipitates in an Al alloy may promote multiplication of dislocations, limit the slip distance, i.e., the mean free path between obstacles, and thus accelerate grain refinement during SPD processing^33–35^. Humphreys *et al*.^36^ noted that the effect of precipitates on grain refinement during SPD depends on the ratio of volume fraction (*F*_*v*_) to diameter (*d)* of the precipitates, i.e., *F*_*v*_/*d*. As mentioned above, in our case, nanosized Fe_3_C precipitates with an average diameter of 70 nm and 90 nm correspond to a *F*_*v*_/*d* of 1.5 × 10^−3^ nm^−1^ and 8.8 × 10^−4 ^nm^−1^. The effect of precipitates on recrystallization can be described in terms of the dislocation-related driving force acting at the grain boundaries and Zener particle-related drag force, as follows^33, 36^:

1 $$F={F}_{c}-{F}_{r}={\rm{0.5}}G{b}^{{\rm{2}}}\rho -3\gamma \frac{{F}_{v}}{d} > {\rm{0}}$$

where *F*_*c*_ is the mean-field driving force acting at an interface, *F*_*r*_ is the Zener drag force induced by precipitate, *F*_*v*_ is the volume fraction of precipitate, *γ* is grain boundary energy, *d* is the diameter of precipitate, *G* is the shear modulus, *b* is Burgers vector, and *ρ* is the stored dislocation density. *F*_*v*_/*d* represents the dispersion ratio of nanosized particles. According to equation (1), increasing *ρ* and decreasing *F*_*v*_/*d* can effectively increase the driving force and promote recrystallization, resulting in improved grain refinement during warm rolling. As illustrated in Figs 2 and 4, the UFG-2 steel includes more grains with HAGBs compared to the UFG-1, though the average grain size of the ferrite is slightly increased from ~430 nm to ~680 nm with decreasing dispersion rate of nanosized Fe_3_C particles from *F*_*v*_/*d* = 1.5 × 10^−3 ^nm^−1^ (UFG-1 steel) to *F*_*v*_/*d* = 8.8 × 10^−4 ^nm^−1^ (UFG-2 steel). During warm rolling processing, numerous dislocations tangles appear around the Fe_3_C precipitates, indicating that the particles are effective obstacles to dislocation motion and thereby contribute to dislocation storage. Hence, Fe_3_C precipitates can significantly facilitate recrystallization and grain refinement through the following two possible mechanisms: (i) The precipitates facilitate retention of high density of dislocations by promoting generation of dislocations and hinder the dislocations from cutting through the precipitates during deformation, which enhances the driving force for recrystallization. (ii) Nanosized Fe_3_C particles modify plastic deformation in their vicinity and thus promote the formation of deformation bands. These regions may act as potential sites for nucleation of new grains.

**Figure 6:**
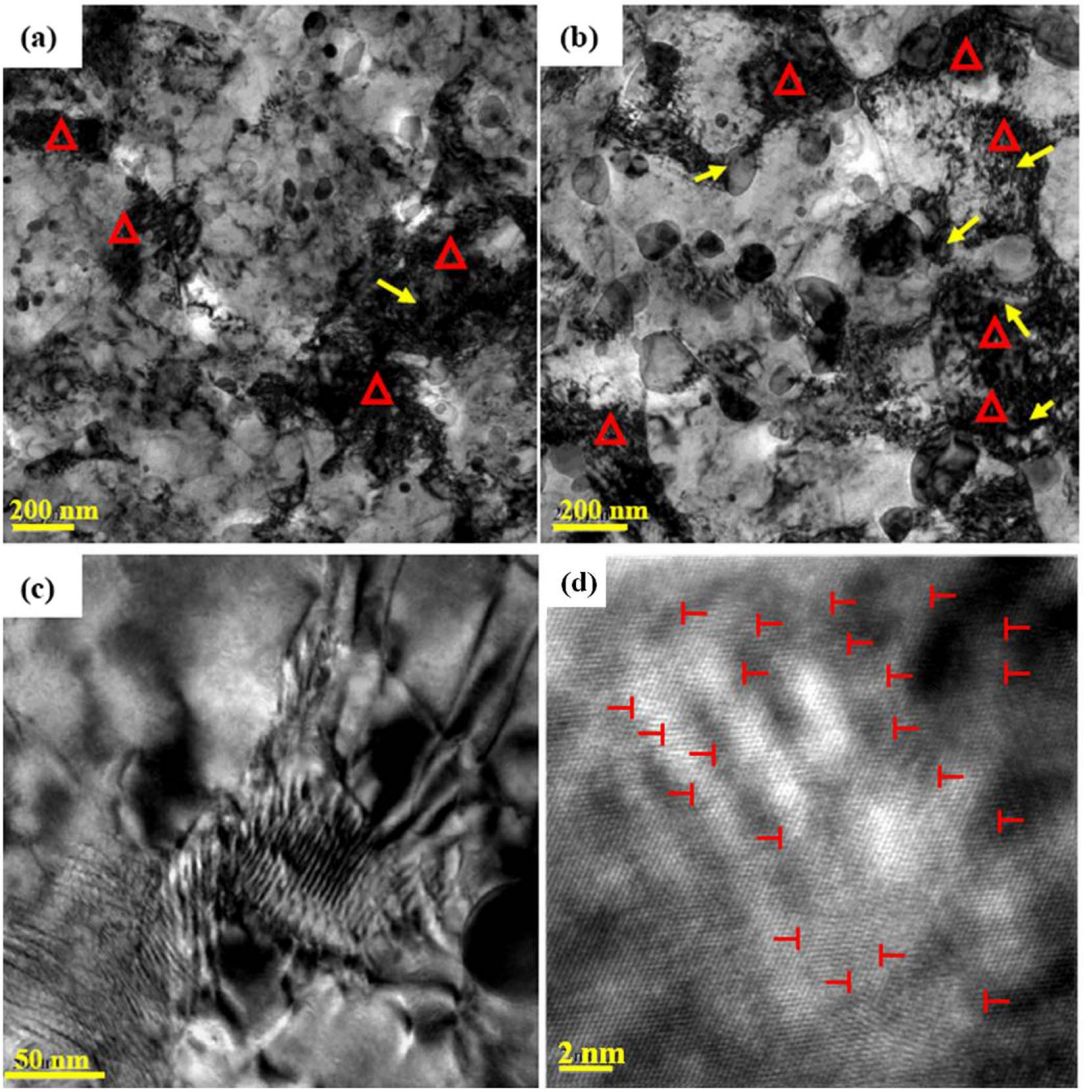
TEM micrographs showing morphology of (**a**) UFG-1 and (**b**) UFG-2 steels after tensile deformation. Some Fe_3_C particles and dense dislocations are indicated by arrows and triangles, respectively. (**c**) Close observation of UFG-2 steel shows dislocations that are accumulated at the edge of nanosized Fe_3_C particles. (**d**) The corresponding HRTEM image of a Fe_3_C particle showing numerous dislocations (indicated as ⊥) are blocked in the interior of the particle.

On the other hand, as the matrix contains particles that are too hard for a dislocation to cut through and bow out, the total dislocation density, *ρ*, can be estimated using the following equation^30^:

2 $$\rho ={\rho }_{{\rm{s}}}+{\rm{\Delta }}\rho ={\rho }_{{\rm{s}}}+6\frac{\dot{\varepsilon }}{b}\frac{{F}_{v}}{d}$$

where *ρ*_*s*_ is the deformation-driven dislocation density of matrix, Δ*ρ is* the increment of dislocation density associated with hard particles, and $\dot{\varepsilon }$ is the shear strain rate. The above deduction can be confirmed by TEM observation in the deformed UFG steels, as shown in Fig. 6. The edge of Fe_3_C particles appear to be extremely blurred because of significant pile-up of dislocations. In both UFG-1 (Fig. 6a) and UFG-2 (Fig. 6b) steels after uniaxial tension, dislocations are blocked at the boundaries of the particles, as indicated by arrows. Here, hard cementite particles act as obstacles to dislocation slip in ferrite, leading to dislocation pile-up at the cementite/ferrite interface. In the case of a finer particle, significantly higher stress is required to make dislocations pass through the particle boundary via cross-slip compared with a larger particle deformed at room temperature. Hence, steels with different particle size, the strength decreases with increasing particle size. Previous atomic simulations^37^ have suggested that the hetero interface within duplex structures provides a strong interatomic bonding strength. Accordingly, numerous dislocations can cut into the brittle phase. In our work, TEM observations clearly indicated that the incoherent interface between nanosized Fe_3_C particles and ferritic matrix acted as a strong barrier to dislocation motion, which led to stress concentration at the edge of the particles (Fig. 6c and d).

The above phenomenon is interesting because it is well accepted that hard particles are effective obstacles to dislocation motion but very few observations directly indicate dislocations in the interior of particles. Generally, grain refinement does not improve the ductility of UFG materials, but inherently poor ductility can be improved by second phase particles. Improved ductility in UFG steel can be attributed to the presence of finely dispersed particles that enhances the work hardening capacity because of accumulation of geometrically necessary dislocations around the particles^10^, as shown in Fig. 6. Numerical simulation of nanoindentation^38^ indicated that the hardness of Fe_3_C particle can be as high as 23.16 GPa at an applied force of 180 nN. The TEM observations clearly indicate that numerous dislocations located within the Fe_3_C particles and dense dislocations are left at the particle/matrix interface, as indicated by triangles in Fig. 6a and b. Hence, our study demonstrates that the contribution of warm rolling and intermittent annealing is a novel approach to obtain ultrafine grains, nanosized precipitates and good ductility. The grain size of low alloy medium-carbon steel is significantly refined to be less than ~1 μm, which is consistent with the recent findings that the nanosized particles may play an important role in the refinement of the matrix^33, 39, 40^. Furthermore, the present study suggests that the nanosized Fe_3_C particles promote plastic deformation of UFG steels by enabling dislocation slip near the particles. It is our hypothesis that there exists an optimum combination of size and volume fraction of ferritic grains and cementite particles, for the desired ductility to be obtained without sacrificing the strength.

## Conclusions

Duplex microstructure composed of ultrafine-grained ferritic matrix and nanosized Fe_3_C particles was obtained in low alloy medium-carbon steel by ingeniously combining multiple pass warm rolling and intermittent annealing. Uniaxial tensile tests and TEM studies were conducted to explore the effect of microstructure on mechanical properties of steels. In UFG-1 steel with the intermittent annealing temperature of 550 °C, *σ*_y_ is as high as 1260 MPa, and *σ*_UTS_ is 1400 MPa. These values are higher than UFG-2 steel with intermittent annealing temperature of 600 °C (*σ*_y_ is 1080 MPa and *σ*_UTS_ is 1200 MPa). The elongation of UFG-1 steel is 15%, while that of UFG-2 steel is 17%. Both the UFG steels show high product of strength and elongation, i.e., the good strength-ductility balance. The good ductility of UFG steels is attributed to the existence of the nanosized Fe_3_C particles. The cementite particles act as strong obstacles for dislocation slip in ferrite. Subsequently, dislocations pile-up at the cementite/ferrite interface, which effectively promotes strain hardening and ductility. The study underscores that the nanosized precipitates not only provide high strength but also contribute to desirable ductility, which is encouraging for improving the ductility of medium-carbon steels.
